# From Lab to Field: CRISPRing Major Cultivated *Solanaceae* for Crop Improvement

**DOI:** 10.3390/ijms27052238

**Published:** 2026-02-27

**Authors:** Martina Ferrero, Alberto Acquadro, Andrea Moglia

**Affiliations:** DISAFA, Department of Agricultural, Forest and Food Sciences, University of Turin, Largo Paolo Braccini 2, 10095 Grugliasco, TO, Italy; martina.ferrero@unito.it (M.F.); alberto.acquadro@unito.it (A.A.)

**Keywords:** *Solanaceae*, genome editing, CRISPR/Cas, protoplasts, VIGE, field trials

## Abstract

The *Solanaceae* family includes some of the most economically and agronomically important crops, such as tomato, potato, pepper and eggplant. Recently, CRISPR/Cas-based genome editing has emerged as a powerful tool for functional genomics and crop improvement, enabling precise and efficient genetic modifications. This review provides an overview of CRISPR/Cas-mediated genome editing technologies and their applications in the major cultivated *Solanaceae* crops. The use of CRISPR/Cas9 systems for targeted gene knockout and knock-in approaches is described, together with advances in precision editing strategies such as base editing and prime editing, which allow precise nucleotide substitutions and small sequence changes. The expanding CRISPR toolbox is further explored through alternative Cas proteins, such as Cas12a and Cas13 with distinct targeting features and potential applications. Emerging delivery strategies, including ribonucleoprotein-mediated editing in protoplasts, virus-induced gene editing (VIGE), de novo induction of meristems and genome editing by grafting, represent promising approaches to generate transgene-free edited plants. In addition, the current status of field trials involving genome-edited *Solanaceae* crops in Europe is outlined, considering the regulatory landscape and legislative requirements for their release in the environment. Despite regulatory constraints, some genome-edited crops have reached the market, highlighting their potential to contribute to sustainable agriculture and crop improvement.

## 1. CRISPR/Cas9-Mediated Genome Editing

In recent years genome editing approaches have emerged as a strategy for efficient and targeted genome modifications, with low costs and a simple and customisable design. These techniques allow several kinds of mutations, such as insertions, deletions, replacements, integration of specific sequences of DNA at a desired genomic locus, and site-directed substitutions across genomes, introducing genetic variation precisely and rapidly [1].

**Figure 1:**
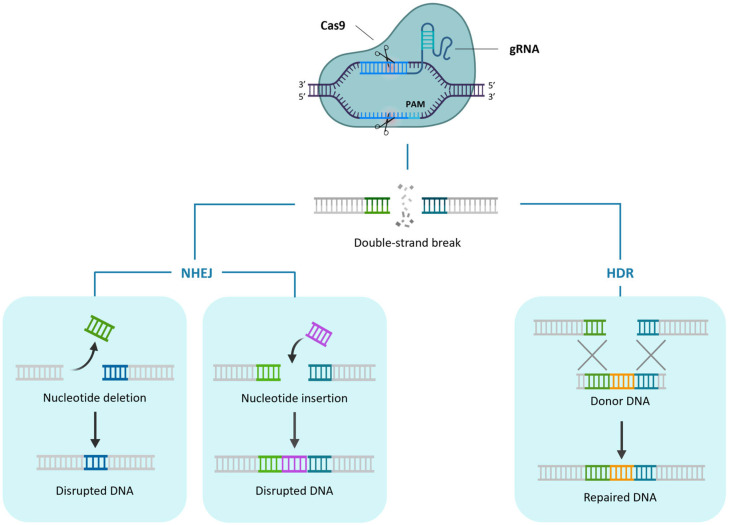
CRISPR/Cas9 system and subsequent DNA repair mechanisms: NHEJ (**left**) and HDR (**right**). (Created in BioRender. Ferrero, M. (2026) https://BioRender.com/o9xj81o).

Genome editing strategies rely on two elements: sequence-specific nucleases, capable of introducing double-strand breaks (DSBs) in the genome at or near the site where a DNA sequence modification is desired, and the cell’s DNA repair pathways. These cellular mechanisms can fix the DSBs through either non-homologous end joining (NHEJ) or homology-directed recombination (HDR). NHEJ frequently generates small insertions or deletions (indels) at target sites, which can lead to frameshift mutations and ultimately result in loss of gene function. HDR uses a donor template as a source of DNA sequence information to repair the DSB by introducing specific point mutations or inserting/replacing desired sequences into the target site (Figure 1) [2].

The clustered regularly interspaced palindromic repeats (CRISPR)/Cas9 system was discovered in archaea and bacteria as an adaptive defence system that serves as a “genetic memory” of prior invasions and allows invading viruses and plasmids to be destroyed. The Cas9 protein recognises the target sequence through base pairing between DNA and a short guide RNA (i.e., crRNA interacting with a tracrRNA with structural function) and then cuts the double-stranded DNA, ultimately avoiding the infection (Figure 1) [3]. The transition of the CRISPR/Cas9 system from biological phenomenon to genome engineering tool came through the discovery that the target sequence could be simply reprogrammed by changing 20 nucleotides in the short guide RNA and that its targeting specificity could be combined with the structural properties of the scaffold RNA in a chimeric single guide RNA (gRNA), thus simplifying the engineering system [4]. This way, Cas9 nuclease activity can be supposedly directed to any DNA sequence of the form 5′-N_20_-NGG-3′, with NGG being the specific protospacer adjacent motif (PAM) recognised by the most widely used Cas9 from *Streptococcus pyogenes* (SpyCas9).

In order to achieve the desired editing outcome and minimise possible side-effects, it is advisable to carry out careful planning, in silico analyses and some validations before conducting a genome editing experiment. The first step is to identify the target gene (or genes) for editing based on the intended phenotypic outcome. Quantitative trait locus (QTL) mapping and genome-wide association studies (GWASs) are the main tools to pinpoint the genetic bases of traits of interest in crop species. After the target identification, it is necessary to retrieve from databases the information about sequences (DNA, mRNA, and coding sequence) of the target gene/genes for the subsequent gRNAs design [5]. Since CRISPR systems’ effectiveness relies on the correct design of gRNAs, it is crucial to select target regions that have a high predicted on-target efficiency while simultaneously minimising off-target risk [6]. A vast number of web-based tools have been developed to assist in this task, implementing prediction models, off-target evaluation and secondary structure prediction [7]. To ensure that the selected gRNAs actually match the genomic sequence of the gene of interest, Sanger sequencing of the target region is recommended [5]. Once it has been verified that the gRNAs’ sequence is correct, an in vitro cleavage assay using DNA amplicons and CRISPR/Cas9 ribonucleoproteins (RNPs) can be a useful validation step to assess the functionality and efficiency of the system on the chosen target [8]. Effective gRNAs should then be inserted into a vector containing the expression cassettes for *Cas9* and a selectable marker gene; several cloning methods are available for the assembly of transformation constructs [9]. If the system is already established in the species of study, it is also possible to perform a protoplast transformation to evaluate the editing efficiency before moving to the final step of stable transformation [5].

CRISPR/Cas9-mediated genome editing may introduce random mutations which are functionally equivalent to spontaneous ones, even though their functional equivalence is not always easy to predict. It has been suggested that most untargeted variations observed in edited lines arise from inheritance from the maternal plants, pre-existing variation in the germline and somaclonal variation during in vitro culture [10]. In *Solanaceae*, several studies have combined whole-genome sequencing (WGS) with targeted deep sequencing of predicted off-target sites in wild-type and CRISPR/Cas9-edited plants. Overall, these analyses support the high specificity of the genome editing system and report no detectable off-target effects [11,12].

The CRISPR/Cas9 system is very versatile and simple to adapt to different purposes, making it the elected strategy for plant genome editing. Indeed, after its discovery, this technique was soon applied to bacteria, yeasts, human cell lines and several model animals, such as fruit flies, nematodes, zebrafish, mice and rats. Simultaneously, CRISPR experiments have been carried out on plants: its efficacy was first demonstrated in model plants such as *Arabidopsis thaliana* and *Nicotiana benthamiana* [13], then in crop plants including rice, wheat, sorghum, maize, soybean, tomato, sweet orange, grape, tobacco and cotton [14]. In fact, CRISPR/Cas9 can play an important role in crop improvement by enabling precise and targeted genetic changes, thus reducing the time required for trait development compared with conventional breeding. In agriculture, CRISPR has been used to enhance yield and quality, improve resistance to pathogens and pests, and increase tolerance to abiotic stresses such as drought, salinity, and heat, supporting the development of more resilient and sustainable crop varieties which require lower inputs for better production [15,16].

Since the *Solanaceae* family includes many of the world’s most important agricultural species (potatoes, tomatoes, eggplants and peppers) and some crop model plants for research (e.g., tomato and tobacco), it is not surprising that several examples of CRISPR/Cas9 applications have involved these species [17]. While efficient protocols have been well established for in vitro regeneration and genetic transformation in tomato and potato, eggplant and bell pepper are still considered as recalcitrant species and are more difficult to handle, with a highly genotype-dependent response to regeneration. This explains the low number of examples in the literature found for CRISPR applications in these two species, if compared to tomato and potato. Indeed, recent studies highlighted the link between bell pepper recalcitrance to in vitro regeneration and the increased production of ethylene; oxidative stress may also be implicated because of browning/necrosis of tissues during in vitro culture [18,19]. Some protocols for regeneration from *Capsicum annuum* have been recently published trying to overcome this recalcitrance through optimisation of the cytokinin content in the media [20], through addition of CaREF1 peptide during regeneration [21] or exploiting morphogenetic regulator genes inducing somatic embryogenesis [22].

### 1.1. CRISPR/Cas9 for Gene Knockout

The first reports demonstrating the feasibility of gene editing in *Solanaceae* species regarded tomato (*Solanum lycopersicum*) and potato (*Solanum tuberosum*), by knocking out the *SlAGO7* [23] and *StALS* [24] genes, respectively, both of them conferring recognisable phenotypes to the edited mutants. Later on, many other applications of CRISPR/Cas9-mediated knockout have been reported in *Solanaceae* crops to improve their performance despite environmental stresses: enhancing disease tolerance through the knockout of either susceptibility genes in tomato (e.g., *SlDMR6-1* [11,25], *SlDND1* [12], *SlPMR4* [26,27], and *SlMLO1* [28]) and potato (e.g., *StPM1* [29], *StDMR6-1* [30,31], *StDND1*, and *StCHL1* [31]) or other genes in potato (e.g., *StDMP2* [32], *StERF3* [33], and *StNPR3* [34]); reducing the impact of viral diseases in tomato (e.g., *eIF4E2* [35], *SlPelo* [36], and CP [37]) and potato (e.g., eIF4E [38,39]); and improving abiotic stress tolerance in tomato (e.g., *SlDMR6-1* [11], *SlCPK28* [40], *SlGRX* [41], and *SlNPR1* [42]) and potato (StDMR6-1 [30]). Genes regulating fruit quality have also been targeted in tomato through CRISPR/Cas9 to improve fruit ripening (e.g., *SlSNAC9* [43] and *SlRIN* [44]), texture (e.g., *SlFIS1* and *SlPL* [45]), and the content of bioactive compounds (e.g., *SlLCYe*, *SlBCH* [46], *SlAPX4* [47], and *Sl7-DR2* [48]). In eggplant (*Solanum melongena*), *DMR6-1* has been knocked out, conferring tolerance to *Phytophthora* spp. [49], and *PPO* genes have also been disabled to reduce fruit browning after cutting [50] and to highlight pleiotropy in this gene family [51]. Potato tuber quality enhancement has been achieved through CRISPR/Cas9-mediated genome editing of genes involved in starch quality (e.g., *StGBSS* [52]), tuber browning (e.g., *StPPO2* [53]), steroidal glycoalkaloids biosynthesis (e.g., *StSSR2* [54] and *St16DOX* [55]) and healthy compound content (e.g., *StbHLH47* [56]). Despite its recalcitrance to genetic transformation and in vitro regeneration, initial results have been recently obtained in pepper (*Capsicum annuum*) by knocking out the functionality of *CaAGO7* and *CaPPO,* conferring, respectively, a visible curly phenotype or a measurable reduction in enzymatic activity [57], and *CaPDS* in a hot pepper accession [58].

Considering that domesticated crops display limited genetic diversity, emerging environmental changes and increasing stresses create an urgent need for novel crops that can better withstand these conditions. However, it is still challenging to exploit wild species for conventional breeding programmes to introgress elite traits in cultivated crops. Multiplexed genome editing approaches (i.e., targeting multiple genes in a single editing event) on genes involved in domestication processes demonstrated the possibility of “reproducing” the genetic events underlying this process and exploiting the wild alleles for crop improvement [59]. Remarkable results have been achieved in tomato by improving the plant architecture, fruit yield and stress resistance thanks to de novo domestication [60,61,62], and similar results were also achieved in another member of the *Solanaceae* family, groundcherry (*Physalis pruinosa*) [63].

Another interesting application of CRISPR/Cas9 technology aims at targeting cis-regulatory elements (CREs) to change the expression of target genes and fine-tune regulatory networks to improve key traits [64,65,66]. In tomato, CREs controlling genes involved in the regulation of interesting traits have been targeted with this strategy. For example, eight gRNAs binding to the promoter of the *SlCLV3* coding sequence generated a wide spectrum of tomato fruit sizes [67]; CRISPR-mediated mutations in the conserved promoter sequences of *SlCLV3* and *SlWUS* were also induced to dissect the impact of cis-regulatory variations on quantitative traits, such as fruit locule number [68]. Similarly, the promoters of *S* and *SP* genes have also been targeted with gRNAs, changing plant and inflorescence architecture [67]. Furthermore, promoter editing was used to create a tomato population with different expression patterns of the *SlWOX9* gene, revealing its pleiotropic functions in vegetative and reproductive meristem development [69]. Another study used CRISPR/Cas9 to engineer allelic variation in the *SlKLUH* promoter around a single-nucleotide polymorphism in a conserved putative CRE associated with fruit weight, causing a consistent increase in fruit weight [70].

### 1.2. CRISPR/Cas9 for Gene Knock-In and Allele Swapping

Compared with the multitude of reports on CRISPR/Cas9-mediated knockout experiments, there are significantly fewer reports on the targeted insertion of DNA segments or allele swapping in plants. This is due to the difficulty in obtaining knock-in events because they require HDR and homologous recombination (HR), which are less prevalent and less efficient in plants compared to NHEJ [71]. Danilo et al. [72] demonstrated the use of HDR to insert some modifications in the tomato *SlALS1* gene. An HDR-mediated allele replacement was performed on the tomato *SlALC* gene to obtain a longer shelf-life of the fruit [73], and even a targeted recombination between homologous chromosomes was achieved [74]. HR was also exploited in combination with geminiviral replicons to repair the CRISPR/Cas9-mediated DBS and restore the wild-type allele of two tomato genes involved in the carotenoid biosynthesis pathway [75]. The same technique was used to insert a strong promoter upstream of a gene controlling anthocyanin biosynthesis in tomato, achieving ectopic anthocyanin accumulation in the tissues [76]. Moreover, HDR was used in potato to re-establish resistance to *Phytophthora infestans* in a susceptible variety by replacing a nonsense mutation in all four alleles of *StCCoAOMT* [77].

Even if *A. tumefaciens*-mediated transformation proved to be very effective for knockout of genes, this method seemed less efficient for knock-ins or allele swapping. Therefore, other delivery methods have been exploited for HDR applications [78]. For example, a geminiviral multi-replicon system was deployed in tomato to increase the HDR-mediated knock-in efficiency, and a salt tolerant allele was obtained by inserting a single aminoacidic substitution in the *HKT1;2* locus [79]. Similarly, herbicide-inhibiting point mutations within the *ALS1* locus have been incorporated in potato, also using a geminiviral replicon [80].

## 2. Base Editing and Prime Editing

Further mutagenesis studies showed that the inactivation of a single nuclease domain converts Cas9 into a nickase (nCas9), whereas simultaneous inactivation of both domains preserves its RNA-guided DNA binding ability while abolishing endonuclease activity, generating the so-called “dead Cas9” (dCas9) [81].

**Figure 2:**
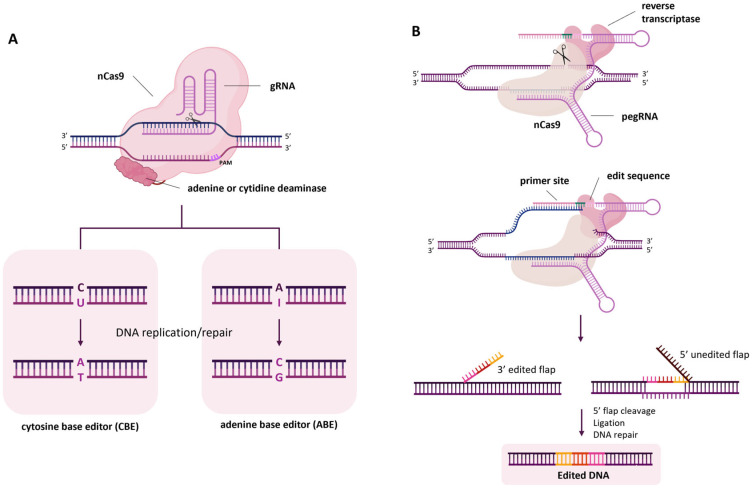
CRISPR/Cas9-mediated base editing (**A**) and prime editing (**B**) systems. (Created in BioRender. Ferrero, M. (2026) https://BioRender.com/mfykcjq).

Base editing allows targeted point mutations to be inserted without DSBs or donor DNA templates. It relies on an nCas9 fused to either an adenine or a cytidine deaminase, leading to an A-to-G (adenine base editor, ABE) or a C-to-T substitution (cytosine base editor, CBE), respectively. The Cas–deaminase complex is guided to the target site by the gRNA and is able to induce a deamination on the non-complementary strand, causing the desired nucleotide substitution (Figure 2A) [82]. This technique was applied at first in tomato, targeting *SlDELLA* or *SlETR1*, two genes involved in plant hormone signalling [83], the *SlALS* gene [84], *SlAGO7* [85] and three genes important for carotenoid accumulation [86]; a transient system has also been used for this purpose in tomato to perform base editing of *SlHWS* [87]. A good efficiency was also achieved in potato on *StGBSS* genes involved in starch synthesis [88,89].

Similarly to base editing, prime editing also uses an nCas9 but fused with a reverse transcriptase. The prime editing guide RNA (pegRNA) includes an sgRNA targeting the specific genomic site, a reverse transcript template (RTT) encoding the desired edit, and a primer binding site (PBS) to initiate the reverse transcription process. The protein complex binds the target DNA and induces a single-strand break where the PBS hybridises to start reverse transcription and eventually to copy the chosen mutation into the genomic DNA (Figure 2B) [90]. This system expands the possibilities for crop improvement by causing essentially every kind of mutation in the desired target site in the genome: indeed, it enables the introduction of indels and all 12 base-to-base conversions (both transitions and transversions). For example, multiple pegRNAs were designed for prime editing of three tomato genes, *SlGAI*, *SLALS2* and *SLPDS1*, and the desired mutations were obtained in the last two, even if with very low efficiencies [91]. To enhance its efficiency in dicotyledonous plants, the prime editing system has been optimised by changing promoters, terminators, nCas and pegRNA variants, including RNA chaperones and using a viral replicon system, obtaining editing efficiencies of up to 38% on the *SlALS* gene [92]. Another high-efficiency Csy4-based prime editing system has been developed for dicots, and in particular tomato, in order to knock in a heat-shock element into the promoter of the *SlLIN5* gene and obtain a better yield under heat stress conditions [93]. Moreover, a dual prime editing system was demonstrated to facilitate precise deletion, replacement and inversion of large DNA fragments in *N. benthamiana* and tomato with high editing efficiency [94]. The prime editing technology also proved to allow successful generation of nucleotide transitions and transversions but also short defined indels in potato [95].

Prime editing shows a favourable safety profile because it avoids double-strand DNA breaks, distinguishing it from conventional CRISPR/Cas9 approaches and generally reducing genomic instability and off-target activity. The activity of prime editing tools at potential sequence-dependent off-target sites was analysed using targeted deep sequencing, and no significant off-target effects were underlined in the transformed tomato lines [92].

To overcome the limited efficiency of prime editing systems, an ultra-efficient prime editing (UtPE) system has been developed for dicotyledonous plants exploiting variants of sixth-generation prime editors (such as PE6c and PE6ec), an altered pegRNA, an RNA chaperone and a geminiviral replicon. This system successfully worked on all tested genomic sites in *Arabidopsis* and tomato, outperforming the previously engineered prime editing systems. Nevertheless, the increases in editing efficiency of the UtPE system did not result in detectable off-target activity, underscoring its precision. Moreover, the desired edited alleles inserted through the UtPE system were stably inherited and caused the desired phenotypic changes [96].

## 3. CRISPR/Cas-Mediated Transcription Activation and Inhibition

**Figure 3:**
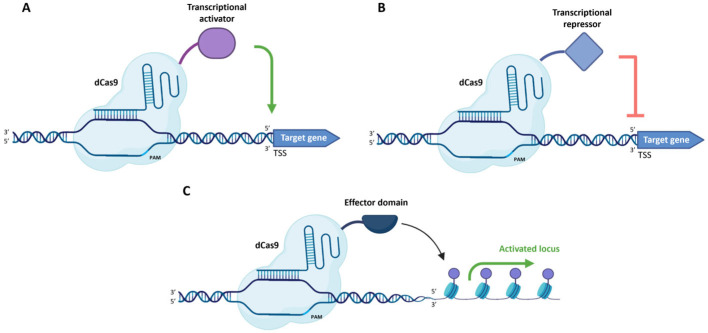
CRISPR/Cas9-mediated transcription activation and inhibition systems: CRISPRa (**A**), CRISPRi (**B**) and epigenetic activation (**C**). (Created in BioRender. Ferrero, M. (2026) https://BioRender.com/y9rge6l).

The inactivation of both the nuclease domains converts the Cas9 into a generic RNA-guided DNA-binding protein that is catalytically inactive (dCas9). It is then possible to directly fuse effectors to the dCas9, transforming the dCas9–effector fusion into an easily programmable artificial transcription factor when paired with a target-specific gRNA [97]. One type of effector that can be fused to dCas9 is a transcriptional activator that will be guided on the desired genomic locus by the complementarity of gRNA and will bind the regulatory region (promoter or enhancer), thus upregulating the expression of the target gene (CRISPRa) (Figure 3A) [98]. Even the modification of epigenetic marks can change the level of transcription of a target gene (Figure 3C).

While most functional genomic studies have relied on the CRISPR/Cas-mediated induction of loss-of-function mutations, gain-of-function approaches offer unique insights [99]. In fact, some examples of applications of CRISPRa have been reported in particular in tomato to obtain upregulation of target genes.

The catalytic domain of the tomato *SlATX1* gene (a histone methyltransferase) was fused to the dCas9 and targeted to the promoter region of the *SlPR-1* gene. Epigenetically edited tomato plants showed enhanced *SlPR-1* transcription that increased biotic stress tolerance and resistance to the pathogenic bacteria *Clavibacter michiganensis* subsp. *michiganensis* [100]. A similar approach has been adopted to target the *SlPAL2* promoter with the same *SlATX1* domain fused to a dCas12; the resulting epigenetic mark caused an upregulation of *SlPAL2* expression in tomato, thus enhancing lignin accumulation and conferring increased resistance to *C. michiganensis* subsp. *michiganensis* without significant pleiotropic effects [101]. Likewise, epigenetic activation through CRISPRa of the *SlWRKY29* gene was found to be linked to changes in chromatic state and somatic embryo induction in tomato, useful to develop a protocol for indirect somatic embryogenesis from cotyledonary explants [102].

A stress-inducible gene regulation has been developed in tomato by fusing the transmembrane domain of a membrane-bound NAC transcription factor with dCas9, so that the complex is sequestered to the plasma membrane under normal conditions and is released in response to heat induction. This way, tomato immunity against bacterial speck disease was enhanced under elevated temperatures by activating *SlCBP60g* and *SlSARD1* genes, and heat-stress tolerance has also been engineered through activation of heat-responsive transcription factors, *SlNAC2* and *SlHSFA6b* [103].

Alternative activation strategies have been developed, such as CRISPR–Act3.0, that proved to be able to recruit additional activators based on the SunTag system and increase activation efficiency of endogenous genes by 240-fold in tomato [104]. MoonTag, another new activator able to recruit the VP64 activation domain, was tested in tomato hairy roots and proved to be more efficient than the older SunTag system in stably activating the expression of the luciferase reporter gene by binding to its synthetic promoter [105].

Another possible application of CRISPR/Cas systems consists in fusing a synthetic transcriptional repressor domain to a dCas9 to induce target gene silencing (Figure 3B). This strategy, known as CRISPR interference system (CRISPRi), offers several advantages over previous forms of gene repression, in particular RNA interference (RNAi) because it does not interfere with the miRNA pathway and has simpler design rules that simplify targeting multiple genes with high levels of knockdown and low off-target effects [97]. Different repressor domains have been tested in plants [106], and an example in potato demonstrated that fusing dCas9 to a KRAB (Krupple-associated box) domain and suppression of the *StSGT1* gene expression through the CRISPRi approach produced plants with altered content of steroidal glycoalkoids, and in particular low α-solanine and unchanged α-chaconine levels [107].

## 4. Alternative Cas Proteins

One of the major limitations of CRISPR/Cas9 systems is the compulsory necessity of a PAM sequence close to the edit site that restricts the target regions to sequences with high GC content (“NGG” for SpyCas9) [108]. Even if some efforts have been made to engineer near-PAMless Cas9 variants [109], very few applications have been reported in plants. For example, an SpCas9-NG variant that recognises an NGN PAM has been engineered and proved to be efficient first in a model plant *Physcomitrella patens* and then also in tomato and potato for both classical CRISPR-generated gene knockout and cytosine base editing [84].

**Figure 4:**
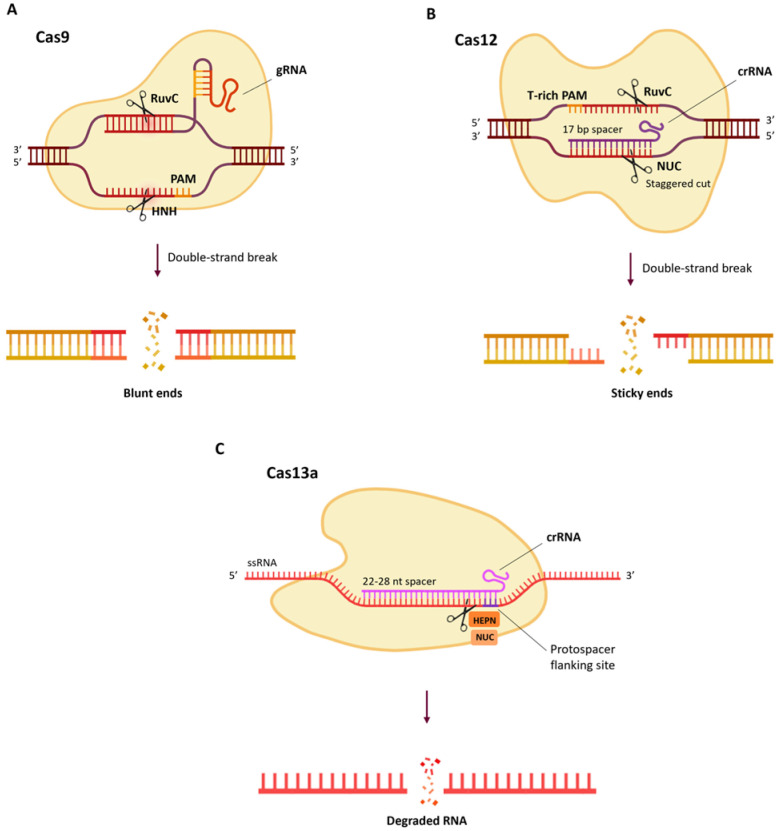
Different Cas proteins and their CRISPR machinery: CRISPR/Cas9 (**A**), CRISPR/Cas12 (**B**), and CRISPR/Cas13a (**C**). (Created in BioRender. Ferrero, M. (2026) https://BioRender.com/0iyc4db).

Exploring the extreme diversity of CRISPR loci [110], many other Cas proteins have been identified with different nuclease activity and different PAM specificity. Among other CRISPR systems, Cas12a (originally identified as Cpf1) may offer a good alternative to Cas9 [111] since it requires “T”-rich PAMs (e.g., TTTV), thus expanding the possibilities for the target region choice. It also generates staggered ended cuts that may promote site-directed integration events through HDR (Figure 4B) [112]. Cas12a variants with altered PAM specificities have also been engineered [113,114]. The efficiency of Cas12a has been tested in tomato protoplasts and resulted similar to Cas9 but with a higher probability of more and larger deletions induced [115]. Vu et al. [79] also proved the efficiency of CRISPR/Cas12a-mediated genome editing in tomato, inserting the *ANT1* gene sequence through HDR in order to obtain visually recognisable anthocyanin-rich tomatoes; the same technique was then applied to the *SlHKT1;2* gene, and a salt-tolerant line was obtained [79]. Further optimisation of the gene targeting system has been performed by using small chemical molecules to inhibit the NHEJ pathway, treating with chemicals such as silver nitrate and selecting high-performance and thermotolerant Cas12a, like the one isolated from *Lachnospiraceae bacterium* [116].

Another Cas protein that can be engineered for knockdown is Cas13. It has RNase activity and when associated with crRNA it forms an RNA-guided RNA targeting complex that recognises and cleaves ssRNA (Figure 4C). This system can be used to target both viral RNA and endogenous mRNA [117]. The CRISPR/Cas13 mechanism is very similar to RNAi, but its high specificity results in low probability of off-target events and high knockdown efficiency. Transgenic potato lines expressing Cas13a/gRNA constructs efficiently suppressed Potato virus Y (PVY) accumulation in the plant and reduced disease symptoms [118,119,120]. Moreover, a CRISPR/Cas13 system targeting with multiple crRNAs the genome of potato spindle tuber viroid (PSTVd) has been applied in tomato plants via transient expression and into *N. benthamiana* through transgenic methods. This resulted in reduced PSTVd accumulation and alleviated disease symptoms [121]. Recently, the knockdown of *PDS* endogenous transcript was demonstrated in *N. benthamiana*, *A. thaliana* and tomato using Cas13a delivered through *Agrobacterium tumefaciens* infiltration. This study also proved that gene silencing can be induced even in the absence of Cas13 by exploiting only the crRNA with *Argonaute* proteins and the plant RNAi machinery [122].

The large size of Cas proteins can represent an obstacle for CRISPR system delivery, especially when the cargo capacity is limited such as in viral vectors. Considering compactness as a sought-after feature, a screening of CRISPR loci has drawn attention to exceptionally small Cas nucleases that can be suitable for manipulating eukaryotic genomes [123]. For example, Cas12f1 isolated from *Syntrophomonas palmitatica* was tested on maize embryos [124], rice calli [125] and *N. benthamiana* leaves [126] with viral vectors, providing efficient editing. Another compact Cas variant called Cas12j or CasΦ proved to efficiently edit target genes in *Arabidopsis* protoplasts [127], soybean hairy roots [128] and *N. benthamiana* leaves [126]. The phage-encoded Casλ was also used to demonstrate editing in *Arabidopsis* and hexaploid wheat protoplasts [129]. Similarly to CRISPR/Cas systems, transposon-encoded proteins nucleases like TnpB [130] and IscB [131] act as reprogrammable RNA-guided DNA nucleases using a small non-coding RNA as a guide to bind and cut DNA. Some of them often share endonuclease domains with Cas proteins, but they are notably smaller [123]. TnpB-based editing systems have been successfully applied to *Arabidopsis* [132,133], rice [134,135] and soybean [136], while IscB has been tested in rice [137].

All the above-mentioned compact editing systems have not yet been applied to solanaceous crops, but they could hold great potential, especially to facilitate the delivery of Cas nucleases through viral vectors because of their small size.

## 5. RNP-Mediated Genome Editing in Protoplasts

**Figure 5:**
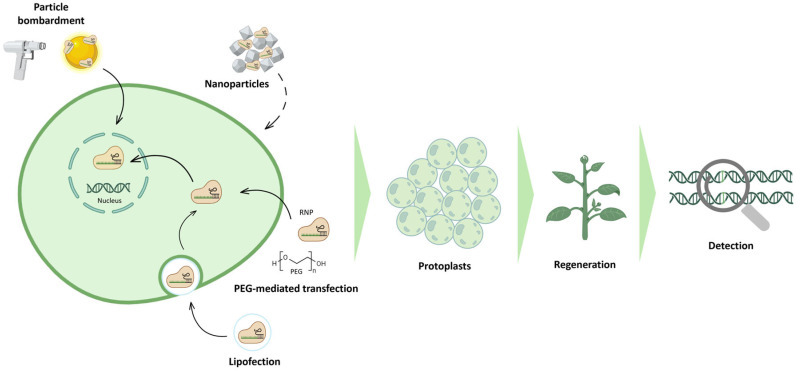
CRISPR/Cas9 RNP-mediated gene editing in protoplasts. CRISPR reagents can be delivered into plant cells as RNPs. Particle bombardment is mainly used to deliver RNPs into explants, while PEG-mediated transfection and lipofection are used for delivery into protoplasts. Nanoparticles are emerging methods for RNP delivery. Transformed cells are then used for plant regeneration and edit detection. (Created in BioRender. Ferrero, M. (2026) https://BioRender.com/5c20hwe).

Most commonly, CRISPR/Cas reagents are delivered into plants as plasmid DNA constructs through *Agrobacterium*-mediated transformation or particle bombardment. In both methods, the plasmid DNA is likely to integrate randomly into one or more genomic loci, resulting in stable and prolonged expression of the CRISPR/Cas components in the host genome [138]. To meet the requirements of the current regulation on genetically modified organisms (GMOs) emerging at the European level, developing gene-edited lines without the integration of foreign DNA into the host genome is gaining increasing importance. Delivery of pre-assembled RNP complexes into protoplasts through either electroporation, polyethylene glycol (PEG) or nanoparticles is a promising alternative way to obtain DNA-free edited plants (Figure 5). The presence of the cell wall in plant cells is the major challenge in delivering the large-sized Cas9 protein along with the negatively charged gRNA; in addition, in vitro regeneration from edited cells is often difficult and highly species-specific. Nevertheless, some achievements have been made in species within *Solanaceae* [139]. Efficient protocols for protoplast isolation and PEG-mediated transfection of RNPs have been established in both the wild tetraploid *Solanum peruvianum*, targeting genes involved in the small interfering RNAs biogenesis (*SpRDR6* and *SpSGS3*), in the pathogen-related peptide precursors (*SpPR-1* and *SpProSys*) and in fungal tolerance (*SpMlo1*) [140], and in the cultivated *Solanum lycopersicum*. In the latter, two genes involved in strigolactone biosynthesis (i.e., *SlCCD7* and *SlCCD8*) were edited, but only calli were regenerated [141]; furthermore, the *SlSP* and *SlSP5G* genes responsible for the control of growth habit and flowering were targeted in tomato protoplasts from four cultivars (namely Red Setter, Alisa Craig, M82 and Moneymaker), and edited plants were regenerated [142]. Another example of application of this technique has been recorded in tomato protoplasts of cv. Heinz 1706, targeting the *SlPelo* gene for tomato yellow leaf curl virus resistance [143]. Similarly, PEG-mediated transfection of potato protoplasts has been optimised and exploited to obtain DNA-free edited plants with lower tuber browning due to *StPPO2* knockout [53,144], modified starch composition by editing of the *StGBSS* gene [52,145] or genes encoding starch branching enzymes [146], and enhanced tolerance to *Phytophthora infestans* by targeting the susceptibility gene *Signal Responsive 4* (*SR4*) [147]. The state of the art is different for eggplant and sweet pepper, which are highly recalcitrant to in vitro regeneration from protoplasts, thus making it more difficult to apply transformation protocols. However, some results have been achieved in pepper with RNP-mediated transfection of protoplasts: gene editing has been highlighted on two different genes, *CaPAD1* or *CaMLO2*, even if with very low editing efficiency, but no plant regeneration was obtained [148,149,150]. No CRISPR/Cas-mediated editing has ever been demonstrated in eggplant protoplasts instead, although a protocol for protoplast isolation and PEG-mediated transfection with vectors encoding fluorescent proteins has been published [151].

Electroporation is another widely employed technique for protoplast transfection with exogenous DNA. A transient electric field applied to the protoplasts induces temporary pores in the cell membrane, allowing the entry of foreign DNA. This method has been mainly exploited for protoplast fusion [152] and transient gene expression [153], but no applications for gene editing have been reported so far in *Solanaceae*.

Nanoparticles can also be used as an alternative delivery method for RNPs: for example, metallic nanoparticles coated with CRISPR/Cas reagents have been used for particle bombardment of rice protoplasts [154], but this strategy is used preferably to penetrate plant tissues or embryos more than cell wall free protoplasts and is used more frequently in monocotyledonous species. It has been demonstrated that addition of anionic polymer polyglutamic acid to standard PEG transfection protocols significantly improved editing efficiencies in *N. benthamiana* protoplasts relative to RNPs alone.

Moreover, a nanoparticle-based platform has been developed to deliver plasmid DNA into *N. benthamiana* using functionalised, high-aspect-ratio carbon nanotube (CNT) nanoparticles, enabling efficient DNA delivery and high levels of protein expression without transgene integration [155]. If combined with CRISPR/Cas tools, this approach could support transgene-free plant genetic engineering. A strategy to conjugate CRISPR/Cas9 machinery to single-walled carbon nanotubes (SWNTs) for cellular delivery in plants has been proposed [156]. This strategy relies on Cas9-SWNT conjugates for RNP delivery, achieved either by direct binding of an engineered Cas9 variant onto the CNT surface or by using a noncovalent peptoid intermediate to bind wild-type Cas9 to the CNT surface. Using peptoid-SWNT delivery of Cas9 RNPs, gene edit was highlighted in the model target gene *PDS* in *N. benthamiana*.

Lipofection seems to be a new promising alternative that allows RNP complexes to be delivered into naked plant cells thanks to cationic lipids that facilitate the interaction between the membranes and RNPs, thus allowing the uptake of the exogenous material [157]. An example of a lipofection-mediated transfection approach for delivery of Cas9/gRNA RNPs into tobacco (*Nicotiana tabacum*) protoplasts has been described by Liu et al. [158], with transfection efficiency reaching up to 66% and a targeted mutagenesis frequency of up to 6%.

## 6. Virus-Induced Gene Editing

Since in vitro regeneration is highly species-specific and sometimes even cultivar-specific [26] and it represents one of the major bottlenecks in plant transformation, alternative strategies have been developed to avoid this step.

**Figure 6:**
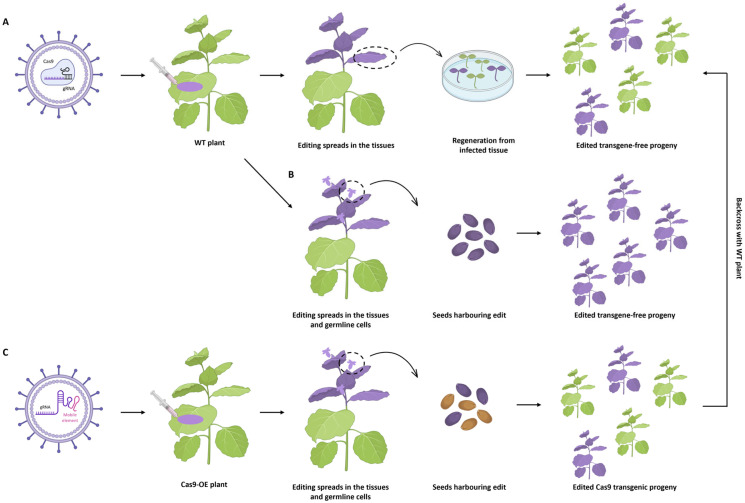
VIGE strategies to obtain heritable gene editing in plants. (**A**) VIGE vectors that deliver both Cas9 and the gRNA spread systemically in the plant. Subsequent tissue culture from infected leaves is needed to regenerate gene-edited plants that can then produce gene-edited Cas-free seeds. (**B**) If the viral infection reaches the meristem, edited seeds and progeny may be obtained. (**C**) VIGE vectors expressing the gRNA fused to mobile elements are used to infect plants that stably express Cas9, thus producing gene-edited Cas9 transgenic seeds. The edited generation needs to be crossed with wild-type plants or self-pollinated to segregate the Cas9 gene. Edited tissues are represented in purple. (Created in BioRender. Ferrero, M. (2026) https://BioRender.com/x440l37).

Virus-induced gene editing (VIGE) is a promising technique in this perspective. It uses viruses as transient delivery vehicles for CRISPR/Cas components and takes advantage of their ability to replicate inside the plant cells to increase accumulation of Cas protein and gRNA within the host cell, ultimately resulting in a higher editing efficiency. To this end, viral genomes are engineered to produce viral vectors encoding CRISPR components able to spread in the plant. VIGE vectors can be divided into two categories according to their cargo capacity and the reagents that may be delivered (Cas9 is a large protein, so it is difficult to fit its long coding sequence in the small viral capsid). The first category includes viral vectors expressing Cas9 and gRNAs that are used to infect wild-type plants. Somatic tissues are edited as the viral infection spreads systemically through the plant. Following tissue culture, infected leaves can be used to regenerate transgene-free edited progeny (Figure 6A). Otherwise, if the infection reaches the meristem, the resulting seeds may also be edited, achieving transgenerational heritability (Figure 6B). In the second category fall viral vectors expressing gRNAs fused to RNA mobility signals, such as *Flowering Locus T* (*FT*) mRNA or transfer RNAs (tRNAs). These are infiltrated into Cas9-overexpressing plants where mobile gRNAs spread systemically, possibly also entering the shoot apical meristem in order to induce editing in the germline. Collected seeds provide edited progeny bypassing tissue culture procedures, but the Cas9 transgene needs to be segregated through backcrossing with wild-type plants or self-pollination cycles (Figure 6C) [159,160]. Viral constructs are usually delivered to the plant through *A. tumefaciens* previously transformed with viral plasmids; agroinoculation is usually done through leaf syringe infiltration, but other procedures can also be used, such as vacuum infiltration, agrospray or agrodrench [159]. The delivery of CRISPR reagents via VIGE was first attempted in *N. benthamiana* using a viral vector based on *Tobacco Rattle Virus* (TRV) [161,162], but many improvements have been implemented to optimise the virus host range, tissue specificity, cargo capacity and efficiency of the system.

Examples of VIGE application in Solanaceous crops have been reported, mainly using *Tobacco Rattle Virus* (TRV), *Potato Virus X* (PVX) or *Tomato Spotted Wilt Virus* (TSWV). Lee et al. [163] were able to knock out the *SlPDS* gene infecting tomato with TRV or PVX and regenerate edited plants by subsequent in vitro culture, and they also demonstrated the applicability of the system in potato and eggplant. By fusing the gRNA targeting *PDS* to the sequence of tomato *FT*, efficient and rapid generation of heritable genome editing was achieved in tomato [164]. Kang et al. [165] showed that low temperatures significantly enhanced VIGE efficiency of TRV and PVX vectors in both cotyledons and systemic upper leaves of tomato, and Oh et al. [166] highlighted the importance of the promoter driving the expression of *Cas9* in order to increase the efficiency of the TRV system and its trans-generational heritability in tomato. PVX has also been efficiently engineered to apply VIGE in tomato, knocking out the *SlPDS* and *SlSGR1* genes [167]. Other functional genes involved in the anthocyanin biosynthesis pathway (namely *SlCHS1*, *SlCHI* and *SlDFR*) have been successfully edited in tomato leaves through TRV-mediated VIGE, and the same technique was also applied on unripe fruits to edit *SlEIL3*, a positive regulator of the ethylene signalling pathway during tomato ripening, thus delaying the ripening in the infiltrated sectors [168]. In addition, TSWV, which has a larger cargo capacity compared to TRV and PVX, has been engineered to deliver CRISPR/Cas9, CRISPR/Cas12a and base editing systems for knockout of different functional genes in *N. benthamiana*; the same system has also been efficiently applied to tomato, chili pepper, sweet pepper and habanero pepper [169]. To improve gRNA mobility in the infected plant, a cleaving hammerhead ribozyme (HH) sequence was added to the TRV2-gRNA construct along with an *FT* mobile RNA or other mobile tRNAs. The editing on *CaPDS* and *CaFA* was successful in *Cas9*-transgenic pepper lines [170]. TSWV was also used in sweet pepper to edit the *PDS* gene via VIGE, and symptomatic leaf tissues from infected plants were used as explants for tissue culture in order to regenerate pepper plants with targeted mutations that were also demonstrated to be germline transmissible [171].

## 7. De Novo Induction of Meristems

**Figure 7:**
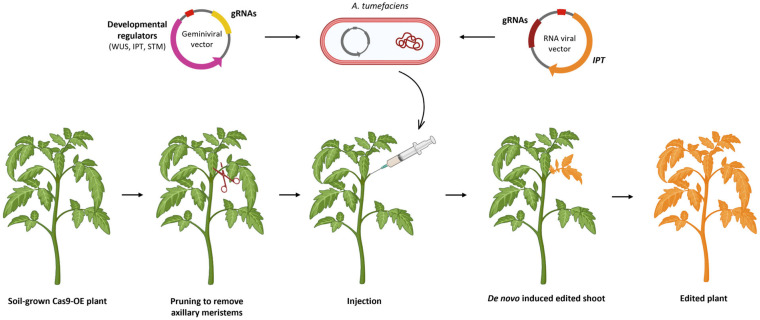
Induction of genetically modified meristems on soil-grown plants. Schematic representation of the procedure to induce genetically modified meristems on soil-grown plants: *A. tumefaciens* culture is perfused in the pruned sites, where DR expression stimulates new gene-edited meristems (orange) to develop into a whole plant. (Created in BioRender. Ferrero, M. (2026) https://BioRender.com/nb5i900).

Another recently developed technique allows tissue culture to be simplified or completely avoided by reprogramming genome-edited somatic cells into meristems through co-expressing developmental regulators (DRs), such as *WUS*, *IPT* and *STM*, and genome editing components, thus enabling direct regeneration of genome-edited plants from somatic cells [172] without requiring dedifferentiation into callus-like structures and then differentiation into shoots and roots. This method enables the creation of either transgenic or gene-edited shoots from de novo induced meristems that proved to be able to produce flowers and seeds, ultimately transmitting transgenes or gene edits to the next generation. Since different combinations of developmental regulators could induce the formation of meristems in different dicot species, the most suitable combination of DRs was first optimised in *N. benthamiana* and then in tomato by delivering constructs expressing different DRs into young seedlings through *A. tumefaciens* coculture. The possibility of generating gene-edited meristems was also demonstrated, transforming seedlings constitutively expressing Cas9 with a vector containing DRs and a gRNA targeting the *PDS* gene [173]. The same constructs were cloned into a T-DNA backbone producing geminiviral replicons derived from bean yellow dwarf virus (BeYDV). These replicons are able to replicate after delivery to plant cells, increasing copy number and leading to high levels of gene expression, even without integration in the genome, potentially generating transgene-free edited lines. Maher et al. [173] also demonstrated that genetically modified meristems could be induced on soil-grown *Cas9*-overexpressing plants by pruning to remove axillary meristems and perfusing the cutting sites with a suspension of *A. tumefaciens* cultures expressing DRs and gRNA cassettes. Gene-edited plants were obtained directly from the resulting shoots, and the induced mutations were inheritable (Figure 7). This protocol has been applied also in tomato and potato, providing a generalisable in planta delivering method that halves the time required for generating gene-edited plants [174]. Similarly, RNA viral vectors expressing gRNAs were delivered to leaves or sites near axillary meristems to induce new edited shoots to form from somatic cells (Figure 7). To enhance the induction of shoots, RNA viral vectors were delivered along with the cytokinin biosynthesis gene *IPT*; this system provided abundant, phenotypically normal, gene-edited shoots from soil-grown tomato plants [175]. This system overcomes the bottleneck in applying VIGE to dicotyledonous crops and reduces the dependency on tissue culture, which can be inefficient in recalcitrant species or cultivars.

Another similar approach to induce transgenic and gene-edited de novo meristems exploits a synthetic cascade made up by a wound-induced transcriptional regulator (*WIND1*) that triggers the expression of DR genes along with gene-editing reagents. This system was applied in planta to non-meristematic internodes of *N. benthamiana* to regenerate de novo shoots with mutations in the *PDS* gene, and *IPT* proved to be the most effective DR among those tested for regeneration. The same synthetic toolkit was successfully applied to both tomato and soybean [176]. Additional DRs were tested at first in snapdragons (*Antirrhinum majus*) and then validated in tomato, highlighting that *PLT5* provided the highest in planta transformation efficiency [177]. The same DR significantly improved the formation of transgenic calli and somatic embryos in sweet pepper through in vitro tissue culture.

Moreover, an *Agrobacterium*-mediated transient expression technology has been applied in tomato to deliver nCas fused to a cytidine deaminase and a viral vector harbouring a gRNA targeting the *WS* gene, involved in the regulation of miRNA expression and plant growth. After agroinfiltration of these reagents, homozygous base-edited plants were obtained, and more than 70% were Cas-free, demonstrating the efficacy of a transient system for base editing that combines different approaches [87].

## 8. Genome Editing Through Grafting

**Figure 8:**
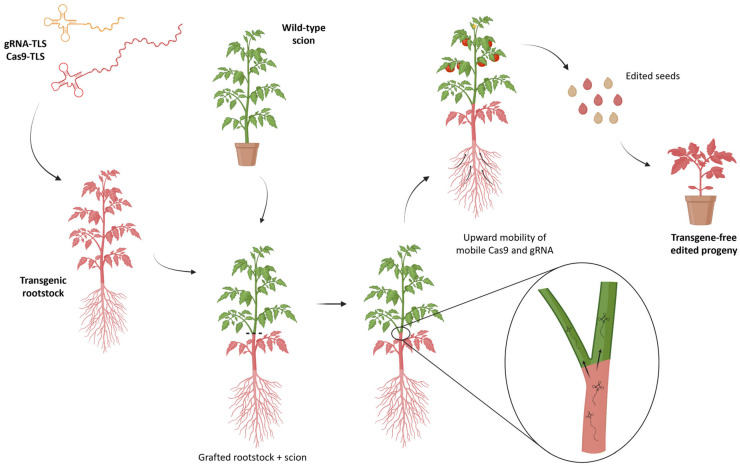
Genome editing through grafting. Schematic representation of the generation of transgene-free edited progeny from a wild-type scion grafted onto a transgenic rootstock expressing Cas9 and gRNAs fused to TLS. Edited tissues are represented in pink. (Created in BioRender. Ferrero, M. (2026) https://BioRender.com/tef9839).

Despite the latest technical advances, delivery systems for genome editing reagents still often depend on the genotype and rely on tissue culture. To overcome these obstacles, an innovative approach has been exploited to develop a transgene-free and heritable strategy for targeted mutagenesis in plants through grafting. The exchange of micro- and macromolecules, such as mobile RNAs, proteins, and hormones, is possible from rootstock to scion or vice versa (Figure 8) [178]. A recent work by Yang et al. [179] showed that Cas9 proteins and gRNAs can move through the grafted junction when fused to a tRNA-like sequence (TLS); this causes an efficient root-to-shoot movement, from a transgenic rootstock to a wild-type scion. The *Cas9* and two gRNAs designed against the *NIA1* gene in *Arabidopsis* were fused to the methionine–tRNA sequence, either intact or truncated, and were expressed in a transgenic *A. thaliana* rootstock. Some weeks after grafting, *Cas9* and gRNA transcripts were detected in the wild-type scion thanks to the long-distance mobility promoted by TLS motifs; some tissues from the grafted scion also developed a visible phenotype with smaller and chlorotic plants because of the inactivation of the target gene. Moreover, *Cas9* and gRNAs transcripts fused to TLS were detected in reproductive tissues from adult grafted plants, and the desired edit was also identified in the offspring, demonstrating the heritability of the system. To demonstrate the possibility of applying this strategy also to more distantly related crops, a *Brassica rapa* scion was heterografted onto a transgenic *Arabidopsis* rootstock, and the presence of both mobile transcripts and gene edits was detected in *B. rapa* siliques and flowers [179].

This kind of genome editing approach could be interesting to implement in *Solanaceae* species that are still difficult to edit because of their recalcitrance to in vitro regeneration. It would be advantageous to produce a transgenic rootstock compatible with grafting with different solanaceous crops (e.g., eggplant and pepper) and to exploit the mobile elements to edit the scion. Furthermore, genome editing through grafting would also be attractive for clonally propagated crops and woody plants that have very limited possibility of improvement through conventional breeding and genetic engineering programmes but are usually grafted to select, maintain or combine desirable traits.

## 9. Regulations for CRISPRed Plants Around the World

In recent years there has been wide debate over whether gene-edited plants should be regulated in the same way as conventionally bred varieties or treated as genetically modified organisms (GMOs). The application of process-based vs. product-based regulatory approaches has been the subject of considerable debate after the emergence of gene-edited plants. As a result, countries have adopted divergent policies [180,181]. The legal framework in each country determines the regulatory status of these plants and, consequently, their prospects for laboratory and field research, as well as the commercialisation (cultivation, import, and export) of their food and feed products. Most regulatory systems distinguish three categories of genome editing using site-directed nucleases (SDNs). SDN-1 generates small sequence changes at the target site, SDN-2 uses a repair template typically via homologous recombination to introduce a specific sequence change, and SDN-3 enables the insertion of larger genetic elements in a template-guided manner, similarly to SDN-2.

According to current legislation, the USA, Canada, Brazil, Argentina, and several Latin American countries (e.g., Chile, Colombia, Paraguay, Honduras, Guatemala, and El Salvador) treat SDN-1 and SDN-2 crops as comparable to conventionally bred varieties. Japan exempts from GMO legislation products coming from SDN-1, SDN-2, and oligonucleotide-directed mutagenesis (ODM), provided that foreign DNA integration can be excluded when relevant. Similarly, Israel classifies gene-edited plants as conventionally bred plants, as long as they do not contain foreign DNA outside the breeder’s gene pool. China has released guidelines for the safety assessment of gene-edited plants that do not contain exogenous DNA sequences (SDN-1 and SDN-2) and employs a tiered evaluation framework based on the risk profile of the targeted trait. India has adopted a risk-based approach and plans to categorise genome editing within the three SDN classes through appropriate risk assessment. The African Union’s Agenda 2063 promotes genome editing as a tool to enhance agricultural productivity and crop resilience; to date, Nigeria and Kenya have introduced case-by-case regulatory review for genome-edited crops. Australia has exempted SDN-1-derived organisms from the more stringent GMO regulatory requirements [180].

However, the regulatory scenario for GM crops varies widely across countries, ranging from relatively permissive frameworks, such as those described above, to more stringent regulatory regimes, like those enforced in New Zealand and the EU. This is the reason why even if a considerable number of gene-edited crops have been produced worldwide, only a few have been approved and even less have made it to the market so far.

The first CRISPR-edited food ever to reach consumers was the ‘Sicilian Rouge’ tomato in 2021, which contains high amounts of γ-aminobutyric acid (GABA). It was developed by Sanatech Seed Co., Ltd. (Tokyo, Japan), a startup from the University of Tsukuba (Japan), by targeting the autoinhibitory domain of the *SlGAD3* gene, whose removal increases the activity of the glutamic acid decarboxylase enzyme that catalyses the decarboxylation of glutamate to GABA [182]. These tomatoes are claimed to have a five- to sixfold increase in GABA content (0.95 mg per gram of fresh weight), thus conferring a health-promoting effect by lowering blood pressure and promoting relaxation through oral intake of GABA, even if no clear evidence is available on this yet [183].

In addition, another CRISPRed product has already been marketed in the USA since 2023 by the biotech company Pairwise, which produced less pungent mustard greens by intervening in the metabolism of the glucosinolates, responsible for the bitter and pungent taste [184]. Furthermore, other genome-edited crops have been approved in different countries but are still waiting for commercialisation: for example, the waxy corn from Corteva Agriscience with high starch content approved in Canada, the non-browning romaine lettuce by Interexon approved in the US, wheat varieties resistant to powdery mildew developed by the Chinese Academy of Sciences approved in China and non-browning bananas by Tropic Biosciences approved in the Philippines.

## 10. Regulation and Field Trials for CRISPRed Plants in Europe

CRISPR technology has revolutionised how and how fast improved crops can be produced, allowing precise modifications in the plants’ genome to control desirable traits. These new traits may help address global agricultural challenges, such as pressures of climate change, food security issues and also producing plant material that meets industrial requirements. Scientific research and laboratory experiments are the starting point to produce new improved varieties, but the plants’ behaviour in a controlled environment must be validated in field conditions to ensure that the desired phenotype is also maintained in a context with multiple stimuli and stresses.

The cultivation of GMOs in the EU was first regulated by the 2001/18/EC Directive [185] that requires a strict risk assessment procedure before authorisation and establishes traceability and labelling criteria for GM products. New Genomic Techniques (NGTs), mainly based on genome editing strategies such as CRISPR/Cas and cisgenesis, have been developed since 2001, thus making the current legislation outdated. Considering that in principle these techniques produce genomic modifications comparable with those achieved through conventional breeding or classical mutagenesis with no foreign DNA introduction in the genomes, NGT products could be suitable for an exemption from the GMO directive [186]. In this view, a new regulatory framework on plants obtained through NGTs was proposed in July 2023 by the European Commission [187]. This proposal distinguishes between NGT category 1 plants, considered equivalent to those produced by conventional breeding or occurring naturally and therefore not subject to the GMO regulation, and all other GM plants, included in NGT category 2 and falling under the 2001 directive. In detail, the criteria of equivalence of NGT-1 plants to conventional plants, as stated in Annex I, are that an NGT product must differ from the parental line by no more than 20 modifications (in predictable DNA sequences) of the following types: (i) substitution or insertion of ≤20 nucleotides; (ii) deletion of any number of nucleotides; (iii) targeted insertion of a contiguous DNA sequence from the breeder’s gene pool or targeted substitution of an endogenous sequence with a contiguous DNA sequence from the breeder’s gene pool, on the condition that this does not result in an intragenic plant; (iv) targeted inversion of a sequence of any size; (v) any other targeted modification, on the condition that the resulting sequence already occur in a species from the breeder’s gene pool [188]. In February 2024, the European Parliament voted in favour of this proposal, and in March 2025 the European Council also supported it with slight changes. After the trilogue phase, on the 4th of December 2025 the council reached a provisional agreement with the European Parliament on a set of rules that establish a legal framework for NGTs that would exempt category 1 plants from GMO requirements [189,190]. On the 28th of January the European Parliament’s Committee on Environment, Public Health and Food Safety (ENVI Committee) voted to approve a deal on New Genomic Techniques (NGTs).

Nevertheless, the experimental release of GM plants is harmonised across the EU through part B of the Directive 2001/18/EC. Following article 6.1 of the directive [185], anyone planning to release GMOs in the environment for experimental purposes must notify the competent authority of the member state in which the release is planned. The notification must contain a technical dossier and an environmental risk assessment and is processed by the authority before giving permission or not for the field trial [191]. This procedure delegates to individual EU states the evaluation and authorisation of field trial requests.

A very different approach has been adopted in the UK after Brexit, with the Precision Breeding Act in 2023 [192] and following Precision Breeding Regulations [193] that entered into full force in November 2025. These regulations introduce the Precision Bred Organisms (PBOs) category that, according to the technical guidance published by the Advisory Committee on Environmental Release (ACRE) [194], includes plants with epigenetic changes, NGT products, and some cisgenic events as long as functional transgenes, such as selectable markers, have been removed (for example, by segregation) and genetic changes introduced could have resulted from traditional processes and selection. Interestingly, in this regulation the decisive point is the nature of the change in the genome, rather than the technique that was used [191], and PBOs can follow simplified procedures for field release after being included in a public national register.

Even though a great number of genome-edited crop plants have already been produced, as reported in peer-reviewed scientific publications and as summarised in the publicly accessible online database managed by the European Sustainable Agriculture Through Genome Editing (EU-SAGE) (https://www.eu-sage.eu/genome-search, accessed on 19 February 2026), in this regulatory landscape only a few examples of NGT plants have been brought to the field for experimentations.

Focusing on *Solanaceae*, three and twelve field trials have been authorised, respectively, for *S. lycopersicum* and *S. tuberosum* in different European countries (Table 1). These events mainly focus on the improvement of crops for their tolerance to biotic and abiotic stresses, for their nutritional value and for their content of industrially useful molecules. In particular, tomato *dmr6-1* mutants [11] with enhanced tolerance to drought stress and *Phytophthora infestans* infection, and *d27* and *ccd7* mutants with higher resistance to broomrapes infestation [195] have been accepted for field trials in Italy, while tomatoes engineered for the accumulation of provitamin D_3_ by genome editing of the *Sl7-DR2* gene [48] have been cultivated for field trials in the UK, and even a clinical trial to test the impact of vitamin D biofortified tomatoes on serum levels of 25(OH)D is ongoing in the UK (https://www.clinicaltrials.gov/study/NCT07142759, accessed on 19 February 2026). Many more NGT events on potato have been accepted for field trials in Northern Europe, particularly dealing with tolerance to late blight, general resistance to pathogens, altered starch composition for industrial purposes and content of antinutritional compounds like glycoalkaloids.

## 11. Conclusions and Perspectives

The advent of NGTs, and in particular CRISPR/Cas technology, has radically changed crop improvement both by making precise modifications possible in desired genetic loci and by speeding up the time required for generating novel improved varieties.

The CRISPR/Cas9 technique (Figure 1) is the first to be developed and is the most used one to produce targeted knockouts in genes linked to desired phenotypes; further optimisations also allowed knock-ins and allele replacements to be produced, even if with a significantly lower efficiency. The arsenal of CRISPR techniques has been expanded with the advent of base editing and prime editing (Figure 2), both exploiting mutagenised Cas9 nickases fused with adenine/cytidine deaminases or a reverse transcriptase, respectively. These methods allow base-to-base substitutions and practically every kind of mutation. By fusing a dCas9 with activator or repressor domains, it is also possible to activate or inhibit the expression of target genes in a precise manner (Figure 3). Other Cas proteins, like Cas12 and Cas13a (Figure 4), have also been isolated and used for gene editing and RNA editing, in parallel with more compact nucleases (such as Cas12f1, CasΦ, Casλ and transposon-encoded TnpB and IscB) that can facilitate delivery because of their small dimensions.

All the above-mentioned techniques usually involve a delivery system that leaves transgenes integrated in the recipient genome, which is undesirable in a transgene-free perspective. Therefore, delivery of RNPs into protoplasts can be a valid alternative guaranteeing the editing without integration of any foreign DNA in the genome (Figure 5). The downside of this technique is that regenerating in vitro from a single naked cell is usually difficult and highly genotype-dependant, thus VIGE can provide a valid alternative by boosting the editing signal through viral vectors agroinfiltrated into somatic tissues (Figure 6). De novo induction of meristems also allows the editing in soil-grown plants to be spread through co-expression of CRISPR reagents with developmental regulator genes (Figure 7), while grafting opens the door to the generation of edited scions through the movement of gRNAs and Cas from a transgenic rootstock (Figure 8).

The list of CRISPR-based technologies has significantly expanded through the years, and the same is true for the available genomic resources, which provide essential support to the development and fine-tuning of genome editing experiments. Nevertheless, a growing limitation to genome editing pipelines based on only one reference genome for the choice of the target gene/genes is that they assume a fixed number of genes and stable gene models. However, *Solanaceae* genomes [196], and generally many plant genomes [197,198], are more dynamic because of recurring genome restructuring and lineage-specific patterns of gene retention, which can generate paralogue contingency. The latter is defined as the dependence of the phenotypic outcome of a certain gene edit on the presence, functionality or expression level of paralogues in the given genetic background [196].

In general, across plants, repeated whole-genome duplications, and subsequent gene loss and lineage-specific retention, generate uneven paralogue repertoires across taxa and backgrounds [199,200]. Similarly, comparative *Solanaceae* pan-genome analyses indicate that ancient polyploidy events can yield divergent sets of duplicated genes across lineages [196]. Moreover, gene families can undergo substantial turnover (duplication and loss) even over a relatively short time, altering redundancy and the probability of compensatory effects. In line with this framework, pan-genetic studies on the *Solanum* genus have shown that duplication and subsequent paralogue diversification can be a major barrier to predictability of phenotypic outcomes and a key determinant for editing results [196].

Interestingly, contingency can be observed within the canonical *CLV*/*WUS* developmental axis. In African eggplant, *Solanum*-wide pan-genetic analyses highlighted that the *CLV3* regulatory circuit reflects lineage-specific paralogue history that can influence meristem and fruit/organ number phenotypes across different genetic backgrounds [196]. This means that the same target gene may sit in different redundancy landscapes depending on which paralogues are present and functional. Therefore, even when the intended edit is molecularly precise and on-target, phenotypic outcomes may cover a wide range of variation, rather than being all-or-nothing. For example, mutations in CREs in tomato’s *CLV*/*WUS* circuitry produced a continuum of fruit/meristem phenotypes [67] that were not simply predictable from alleles or changes in gene expression, thus complicating expectations about the outcome of gene edits. These issues are especially relevant for loci controlling meristem architecture and fruit size, where paralogue-rich families can show differential retention and functional partitioning across *Solanum* lineages [196]. In practice, this means that a gRNA designed using a single reference genome may: (i) miss non-reference alleles or presence/absence variations; (ii) unintentionally target multiple paralogues in different accessions; (iii) produce different outcomes across genotypes because some backgrounds carry extra functional paralogues that can compensate.

Overall, genome editing in *Solanaceae* should increasingly rely on pan-genome and graph-based references. Such resources are now available for multiple major crops in the family, including eggplant [201,202], tomato [203] and potato [204], enabling structural and gene-content diversity to be incorporated into target selection, improving the robustness of editing outcomes.

The ultimate objective of crop improvement programmes is to introduce new crop cultivars to the market that can better respond to the challenges that climate change and growing population create; genome editing strategies, and in particular NGTs, offer faster and more cost-effective solutions to this matter. Nevertheless, the highly diversified legislative context across the world makes it difficult to adopt a clear stance towards genome editing products, but of course the hope is that more and more CRISPRed foods will get approval, reach the fields and ultimately the market to help fight the challenges that global agriculture faces. Alongside, the regulatory frameworks need to evolve, particularly in the countries with a more stringent regulation, in order to adapt to the NGTs and their products.

## Figures and Tables

**Table 1:** Authorised field trials in Europe for NGT varieties in *Solanaceae* species.

Species	Variety	Gene	Nation	Notification Number	Trait
*S. lycopersicum*	San Marzano	*DMR6-1* [11]	Italy	B/IT/25/03	Tolerance to abiotic and biotic stress
*S. lycopersicum*	Alisa Craig	*D27*, *CCD7* [195]	Italy	B/IT/24/02	Reduced strigolactone resistance to *Orobanche* spp.
*S. lycopersicum*	MoneyMaker	*7-DR2* [48]	UK	23/Q06	Conversion of provitamin D_3_ to vitamin D_3_ in sunlight
*S. tuberosum*	Ydun	*DMR6-1*	Denmark	B/DK/24/23110	Tolerance to late blight
*S. tuberosum*	Ydun	*DMR6-1*	Denmark	B/DK/54/StDMR6-1 LoF Ydun	Altered sensitivity to blight
*S. tuberosum*	Wotan	*GBSS1*	Denmark	B/DK/55/Waxy Wotan	Amylopectin-only starch
*S. tuberosum*	Various	*Rpi-chc1*, *Rpi-cap1*, *ELR1*, *Peru*/*DND1*, *PMR4*, *DMR6-1*, *SR4*	Netherlands	B/NL/25/007	Increased resistance/lower susceptibility to *Phytophthora infestans*
*S. tuberosum*	Kuras	*DMR6-1*	Denmark	B/DK/25/Kuras	Tolerance to late blight
*S. tuberosum*	Ydun	*CBP-1*	Denmark	B/DK/25/Ydun_v1	Tolerance to late blight
*S. tuberosum*	NA	*GBSS*, *SSS3*, *SSS2*	Sweden	B/SE/19/5614	Lack of amylose starch
*S. tuberosum*	NA	*DMR6*, *CHL1* [31]	Sweden	B/SE/21/3359	Altered resistance to pathogens
*S. tuberosum*	NA	*CHL1* [31]	Sweden	B/SE/20/1726	Altered resistance to pathogens
*S. tuberosum*	NA	*DMR6-1* [31]	Sweden	B/SE/23/3093	Altered resistance to pathogens
*S. tuberosum*	Various	*GBSS* [52], *SSS*, *SBE* [147]	Sweden	B/SE/22/23780	Tailored starch quality
*S. tuberosum*	Various	*GBSS*, *SSS3*, *SSS2*, *SSS1*, *SSS4*, *SSS5*, *SSS6*, *SBE1*, *SBE2*/*RMA1H1*, *SMO1*, *DWF1*, *GAME9*, *DWF7*, *GAME6*, *GAME11*, *GAME4*, *GAME12*/*DMR6*, *Parakletosis*, *eIF4E*	Sweden	B/SE/24/20422	Altered starch quality/decreased content of antinutritional compounds (TGA)/reduced susceptibility to late blight and PVY

## Data Availability

Data sharing is not applicable. No new data were created or analysed in this study.

## Abbreviations

The following abbreviations are used in this manuscript:

CRISPR	Clustered Regularly Interspaced Short Palindromic Repeats
DNA	Deoxyribonucleic acid
RNA	Ribonucleic acid
Cas	CRISPR associated
PAM	Protospacer adjacent motif
GMO	Genetically modified organism
